# Supplementary material for: The causal relationship between the human gut microbiota and pyogenic arthritis: a Mendelian randomization study
Source: Front Cell Infect Microbiol. 2024 Nov 26;14:1452480. doi: 10.3389/fcimb.2024.1452480 (PMC11629706; doi:10.3389/fcimb.2024.1452480)
Supplement: Supplementary file 1 [file DataSheet1.zip › mendelian test/ebi-a-GCST90027585.csv_forest.pdf]

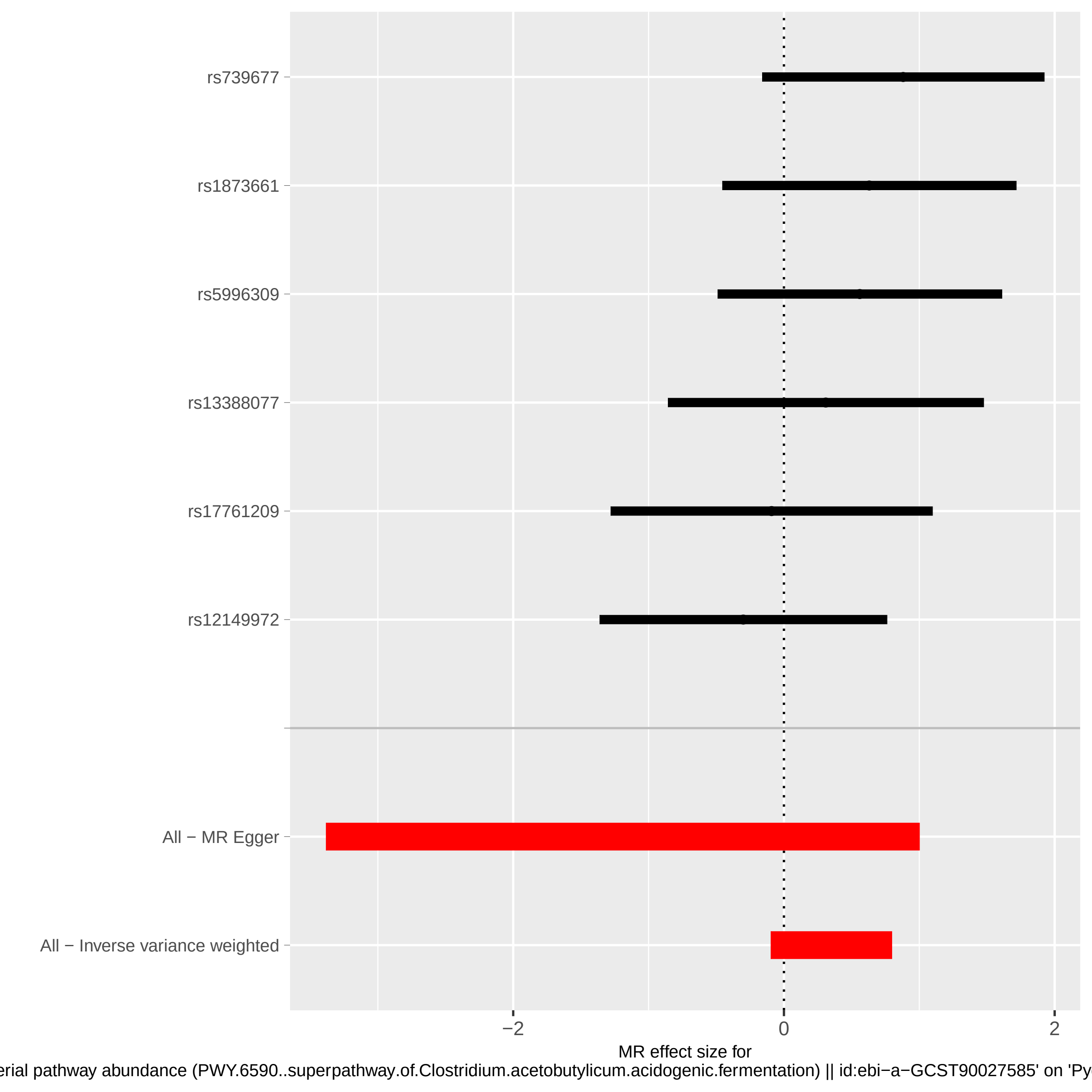

rs739677
rs1873661
rs5996309
rs13388077
rs17761209
rs12149972
All − MR Egger
All − Inverse variance weighted
−2
0
2
MR effect size for
erial pathway abundance (PWY.6590..superpathway.of.Clostridium.acetobutylicum.acidogenic.fermentation) || id:ebi−a−GCST90027585' on 'Py
